# Sexually transmitted infections and other health issues among transgender women and travestis in Brazil: epidemiological profile, vulnerabilities, access to services and care

**DOI:** 10.1590/1980-549720240001.supl.1

**Published:** 2024-08-19

**Authors:** Maria Amélia de Sousa Mascena Veras, Inês Dourado, Francisco Inácio Bastos, Thiago Félix Pinheiro

**Affiliations:** I Santa Casa de São Paulo, School of Medical Sciences – São Paulo (SP), Brazil.; II Núcleo de Pesquisa e Direitos Humanos em Saúde da População LGBT+ – São Paulo (SP), Brazil.; III Universidade Federal da Bahia, Institute of Collective Health – Salvador (BA), Brazil.; IV FundaçãoOswaldo Cruz, Institute of Scientific and Technological Communication and Information on Health – Rio de Janeiro (RJ), Brazil.

Sexually Transmitted Infections (STIs) are a public health issue worldwide, presenting high rates of morbidity and mortality and having a significant impact on quality of life, especially in the most vulnerable population segments such as Transgender Women and *Travestis* (TGW)^1^. Despite the scarcity of data on STIs in this population group, especially in low- and middle-income countries, such as Brazil, existing studies, generally related to HIV, indicate that this is one of the most affected populations.

In this sense, the TransOdara study aimed to estimate the prevalence of the most relevant STIs among TWT in Brazil and to investigate issues related to illness and the search for care, in order to produce knowledge of their health conditions and psychosocial aspects, as well as to foster improvements in their relationship with healthcare services. This study was designed and implemented in partnership with public research institutions in the different macro-regions of the country and it was funded by the Brazilian Ministry of Health and the Pan American Health Organization (PAHO).

TransOdara was the name proposed by a group of TWT specially gathered to discuss the visual identity of the study. The initial portion of the name specifies the target population for the research and indicates its aim to encompass a wide range, including regional, of identity nominations. In Brazil, the term “trans” has been employed as a broad term for individuals with gender identities and expressions that do not align with their sex assigned at birth. The research specifically examined the segment of this population that identifies with the female spectrum. In turn, the term ‘Odara,’ of Yoruba origin, was incorporated into *pajubá*, a “dialect” developed by Brazilian *travestis2*, expressing connotations of positivity, beauty, prosperity, and wonder (as in the famous Caetano Veloso’s music), the term translates the participants’ expectations regarding the study.

Conducting research with the transgender population, from the perspective of social justice and the universal right to health^3^, calls for a dialogue with its questions and claims, as well as to foster the implementation of the results in concrete interventions capable of providing advances and answers. In this sense, despite the challenges posed by the simultaneous occurrence of the new coronavirus (COVID-19) pandemic and the fieldwork targeting a vulnerable and spatially dispersed population^4^, the study produced important data for the knowledge of the health conditions of TWT in Brazil and to inform public policies specific for this population. At the same time, it has structured, together with the partner healthcare services, the provision of qualified, agile, and attentive care to the health issues of this population in five Brazilian capitals: Manaus, Salvador, Campo Grande, Porto Alegre, and São Paulo.

This special issue presents the main results of the research in its quantitative-qualitative approach, aiming to foster the debate on health issues among TWT. In the first article, the authors describe the methodological bases of TransOdara, its design, and the collaborative efforts of different teams of researchers, in addition to characterizing the participants in relation to sociodemographic and behavioral aspects. Authors of the following articles provide the prevalence of the investigated STIs among TWT, namely: syphilis^5^, HIV^6^, viral hepatitis^7^, chlamydia and^8^. In short, they present prevalences or exposure markers higher than those estimated for the general population in Brazil and demonstrate that access to vaccines or other prevention technologies is low, underlining the context of TWT vulnerability and pointing out individual and socio-behavioral factors associated with such infections.

In addition, authors of another article^9^ reiterate the difficulties faced by TWT in the search for health care, but also point to a positive ongoing change through the expansion of the availability of services and the improvement of care. The article on testing for STIs^10^ highlight the need to expand access and bonding of TWT to healthcare services to reduce the transmission of HIV and other STIs. Conversely, authors of the article on the acceptability of physical examinations for the detection of STIs^11^ explain that a gender affirmation perspective should be incorporated to carry out physical examinations with TWT.

A last set of articles refer to other relevant topics to understand the relationship of TWT with health, illness, and care processes. The first^12^ demonstrates that the use of nonprescription hormones is still very high, showing limitations in access to care related to gender transition. In another^13^, the authors observed an intense and diversified use of psychoactive substances, associated with other social markers of difference such as unstable housing. Authors of another article^14^ present a high proportion of gender identity discrimination among TWT, associated with greater vulnerability and history of violence. In another article^15^, more than half of the study participants had already been victims of sexual violence, a phenomenon also associated with greater socioeconomic vulnerability including having no place to live; still, most did not seek help from healthcare services. Lastly^16^, an article addressed the experience of incarceration and found a relatively high proportion of TWT who were deprived of liberty, a situation in which they report having suffered physical, sexual, and moral violence.

We hope that the publication of these results will contribute to boosting the STI prevention agenda for TWT, drawing the attention of all those interested in the subject, including public health authorities, to the specific needs of this population group.
