# Supplementary material for: Sexually transmitted infections and other health issues among transgender women and travestis in Brazil: epidemiological profile, vulnerabilities, access to services and care
Source: Rev Bras Epidemiol. 2024 Aug 19;27(Suppl 1):e240001.supl.1. doi: 10.1590/1980-549720240001.supl.1 (PMC11338530; doi:10.1590/1980-549720240001.supl.1)
Supplement: Translation

# Translation: Infecções sexualmente transmissíveis e outras questões de saúde de mulheres trans e travestis no Brasil: perfil epidemiológico, vulnerabilidades, acesso a serviços e cuidado

**DOI:** 10.1590/1980-549720240001.supl.1.2

**Authors:** Maria Amélia de Sousa Mascena Veras, Inês Dourado, Francisco Inácio Bastos, Thiago Félix Pinheiro

**Affiliations:** I Santa Casa de São Paulo, Faculdade de Ciências Médicas – São Paulo (SP), Brasil.; II Núcleo de Pesquisa e Direitos Humanos em Saúde da População LGBT+ – São Paulo (SP), Brasil.; III Universidade Federal da Bahia, Instituto de Saúde Coletiva – Salvador (BA), Brasil.; IV Fundação Oswaldo Cruz, Instituto de Comunicação e Informação Científica e Tecnológica em Saúde – Rio de Janeiro (RJ), Brasil.

As Infecções Sexualmente Transmissíveis (ISTs) constituem um problema de saúde pública em todo o mundo, apresentando índices elevados de morbidade e mortalidade e tendo impacto significativo na qualidade de vida, sobretudo dos segmentos populacionais mais vulnerabilizados, como é o caso das Mulheres Trans e Travestis (MTT)^1^. Apesar da escassez de dados sobre ISTs nesse grupo populacional, especialmente em países de renda baixa e média, como o Brasil, os estudos existentes, em geral relativos ao HIV, apontam que essa é uma das populações mais afetadas.

Nesse sentido, o estudo TransOdara objetivou estimar a prevalência das principais ISTs entre MTT no Brasil e investigar questões relacionadas ao adoecimento e à busca por cuidados, com o intuito de produzir conhecimento acerca de suas condições de saúde e de aspectos psicossociais, bem como de fomentar melhorias em sua relação com os serviços de saúde. Este estudo foi planejado e desenvolvido em parceria com instituições públicas de pesquisa das diferentes macrorregiões do país e contou com financiamento do Ministério da Saúde e da Organização Pan-americana de Saúde (OPAS).

TransOdara foi o nome proposto por um grupo de MTT especialmente reunidas para discutirem a identidade visual do estudo. A primeira parte do nome demarca a população à qual a pesquisa foi destinada e expressa seu intuito de abranger a diversidade (regional, inclusive) de construções identitárias. O termo *trans* tem sido utilizado no Brasil como categoria englobante das pessoas com identidades e expressões de gênero que diferem do sexo atribuído a elas no nascimento. O estudo foi circunscrito à parte dessa população que se identifica no feminino. Já *odara*, termo de origem iorubá, presente no contexto da religiosidade afro-brasileira, passou a ser utilizado no *pajubá*, “dialeto” desenvolvido por travestis brasileiras^2^. Com o significado de bom, bonito, próspero, maravilhoso (tal como aparece na música de Caetano Veloso), indica a expectativa das participantes em relação ao estudo.

Fazer pesquisa com a população trans, na perspectiva da justiça social e do direito universal à saúde^3^, convoca à interlocução, com suas questões e reivindicações, bem como à efetivação dos resultados em intervenções concretas capazes de proporcionar avanços e respostas. Nessa direção, apesar dos desafios acarretados pela ocorrência simultânea da pandemia da COVID-19 e do trabalho de campo dirigido a uma população vulnerabilizada e dispersa espacialmente^4^, o estudo produziu dados importantes para o conhecimento das condições de saúde de MTT no Brasil e para a orientação de políticas públicas específicas. Ao mesmo tempo, estruturou junto aos serviços de saúde parceiros a oferta de uma assistência capacitada, ágil e atenta às questões de saúde dessa população em cinco capitais brasileiras: Manaus, Salvador, Campo Grande, Porto Alegre e São Paulo.

Este suplemento apresenta os principais resultados da pesquisa em sua abordagem quantitativa-qualitativa, visando fomentar o debate sobre agravos em saúde entre MTT. No primeiro artigo, os autores descrevem as bases metodológicas do TransOdara, seu desenho e os esforços colaborativos de diferentes equipes de pesquisadores, além de caracterizar as participantes em relação aos aspectos sociodemográficos e comportamentais. Os artigos seguintes estimam entre MTT a prevalência das ISTs investigadas: sífilis^5^, HIV^6^, hepatites virais^7^ e clamídia e gonorreia^8^. Em síntese, apresentam prevalências ou marcadores de exposição mais altos do que os estimados para a população geral no Brasil e demonstram que o acesso a vacinas ou a outras tecnologias de prevenção é baixo, sublinhando o contexto de vulnerabilidade de MTT e apontando fatores individuais e sociocomportamentais associados a tais infecções.

Adicionalmente, outro artigo^9^ reitera as dificuldades enfrentadas por MTT na busca por cuidado à saúde, mas também capta a uma mudança positiva em curso por meio da ampliação da disponibilidade de serviços e da melhoria da assistência. O artigo sobre testagem para ISTs^10^ destaca a necessidade de ampliar o acesso e a vinculação de MTT aos serviços de saúde para reduzir a transmissão do HIV e de outras ISTs. Já o artigo acerca da aceitabilidade de exames físicos para detecção de ISTs^11^ explicita que uma perspectiva da afirmação de gênero deve ser incorporada para realizar exame físico com MTT.

Um último conjunto de artigos se refere a outros temas relevantes para compreender a relação de MTT com os processos de saúde, adoecimento e cuidado. O primeiro^12^ demonstra que o uso de hormônios sem prescrição é ainda muito alto, evidenciando limitações de acesso aos cuidados relacionados à transição de gênero. Em outro^13^, os autores observam um uso intenso e diversificado de substâncias psicoativas, associado a outros marcadores sociais de diferença, como moradia instável. Um artigo^14^ apresenta uma alta proporção de discriminação por identidade de gênero entre MTT, associada a maior vulnerabilidade e histórico de violência. Ainda, em outro artigo^15^ mais da metade das participantes do estudo já foram vítimas de violência sexual, fenômeno também associado à maior vulnerabilidade socioeconômica e a não ter onde morar; ainda, a maioria não buscou auxílio em serviços de saúde. Um último artigo^16^ abordou a experiência de encarceramento e encontrou uma proporção relativamente alta de MTT que foram privadas de liberdade, situação na qual denunciam ter sofrido violência física, sexual e moral.

Esperamos que a publicação desses resultados contribua para impulsionar a agenda de prevenção das ISTs em MTT, chamando a atenção de todos/as os interessados/as no tema, incluindo autoridades de saúde pública, para as necessidades específicas desse grupo populacional.
